# Supplementary material for: A subset of type 4 secretion system effectors of Brucella spp. associates to outer membrane vesicles
Source: Microlife. 2026 Jan 2;7:uqaf046. doi: 10.1093/femsml/uqaf046 (PMC12794620; doi:10.1093/femsml/uqaf046)
Supplement: uqaf046_Supplemental_Files [file uqaf046_supplemental_files.zip › supplementaryinformation.pdf]

Supplementary Information

Table S1: Strains used in this study.

Strain	Relevant characteristics	Reference/Source	Description
Escherichia coli			
Stellar	*F-, endA1, supE44, thi-1, recA1, relA1, gyrA96, phoA, Φ80d lacZΔ M15, Δ(lacZYA-argF) U169, Δ(mrr-hsdRMS-mcrBC), ΔmcrA, λ-*	Takara bio	Standard cloning strain
JKE201	MFDpir Δ*mcrA* Δ*(mrr-hsdRMS-mcrBC) aac(3)IV::lacIq*	(Harms *et al.* 2017)	Donor strain for conjugation
Brucella abortus			
	Brucella abortus 544	J-M. Verger, INRA, Tours	
RCBr01	*Brucella abortus* 2308	Kind gift of J. P. Gorvel, Centre d'Immunologie de Marseille-Luminy (CIML) (FR)	
RCBr03	*Brucella abortus* 2308 Δ*virB9*	Kind gift of J. Celli, Washington State University (USA) (Celli, Salcedo and Gorvel 2005)	
MKBr144	*B. abortus* p*bspC-hibit*	This study	
MKBr146	*B. abortus* Δ*virB9* p*bspC-hibit*	This study	
MKBr150	*B. abortus* pha.*bspC*$_{\Delta SP}$*-hibit*	This study	
MKBr156	*B. abortus* p*bpe123-hibit*	This study	
MKBr160	*B. abortus* p*vceA-hibit*	This study	
MKBr162	*B. abortus* Δ*virB9* p*vceA-hibit*	This study	
MKBr164	*B. abortus* p*vceA*$_{\Delta SP}$*-hibit*	This study	
MKBr138	*B. abortus* p*p17-hibit*	This study	
MKBr152	*B. abortus* p*eipA-hibit*	This study	
MKBr166	*B. abortus* p*bspL-hibit*	This study	
MKBr168	*B. abortus* Δ*virB9* p*bspL-hibit*	This study	
Brucella microti			
MKBMi103	*Brucella microti* CCM 4915T	Kind gift of Sascha Al Dahouk,<br>Product Hygiene and Disinfection Strategies at Bundesinstitut für Risikobewertung Berlin (GER)	
MKBMi139	*B. microti* CCM 4915T Δ*virB9*	This study	
MKBMi128	*B. microti* p*phoA*$_{\Delta SP}$	This study	
MKBMi251	*B. microti* Δ*virB9* p*phoA*$_{\Delta SP}$	This study	
MKBMi130	*B. microti* p*SP*$_{phoA}$-*phoA*$_{\Delta SP}$	This study	
MKBMi252	*B. microti* Δ*virB9* p*SP*$_{phoA}$-*phoA*$_{\Delta SP}$	This study	
MKBMi134	*B. microti* p*bspD-phoA*$_{\Delta SP}$	This study	
MKBMi144	*B. microti* p*bspC-phoA*$_{\Delta SP}$	This study	
MKBMi253	*B. microti* Δ*virB9* p*bspC-phoA*$_{\Delta SP}$	This study	
MKBMi145	*B. microti* p*SP*$_{bspC}$-*phoA*$_{\Delta SP}$	This study	
MKBMi254	*B. microti* p*bspC*$_{\Delta SP}$-*phoA*$_{\Delta SP}$	This study	
MKBMi255	*B. microti* p*vceA-phoA*$_{\Delta SP}$	This study	
MKBMi256	*B. microti* Δ*virB9* p*vceA-phoA*$_{\Delta SP}$	This study	
MKBMi257	*B. microti* p*SP*$_{vceA}$-*phoA*$_{\Delta SP}$	This study	

MKBMi258	*B. microti* p*vceA*$_{\Delta SP}$-*phoA*$_{\Delta SP}$	This study
MKBMi229	*B. microti* p*p17-phoA*$_{\Delta SP}$	This study
MKBMi230	*B. microti* p*ttpA-phoA*$_{\Delta SP}$	This study
MKBMi231	*B. microti* p*eipA-phoA*$_{\Delta SP}$	This study
MKBMi148	*B. microti* p*bspC-hibit*	This study
MKBMi171	*B. microti* p*bpe123-hibit*	This study
MKBMi181	*B. microti* p*vceA-hibit*	This study
MKBMi224	*B. microti* p*p17-hibit*	This study

Table S2: Plasmids used in this study.

Plasmid	Description	Reference
pNPTS138	Cloning suicide vector encoding *sacB*, Kan^R	Kind gift of U. Jenal, Biozentrum of the University of Basel (CH)
pNPTS138_Δ*virB9*$_{BMi}$	Suicide vector for in-frame deletion of *virB9* from *B. microti*	This study
pJS140	pBBR-derivative with pTetAR2, dsRed under P_{aphT}, Cm^R	(Ketterer *et al.* 2024)
p*phoA*$_{\Delta SP}$	Derivative of pJS140, aTc-inducible expression of Flag-PhoA$_{\Delta SP}$	This study
p*SP*$_{phoA}$-*phoA*$_{\Delta SP}$	Derivative of p*phoA*$_{\Delta SP}$, aTc-inducible expression SP$_{PhoA}$- Flag-PhoA$_{\Delta SP}$	This study
p*bspC*-*phoA*$_{\Delta SP}$	Derivative of p*phoA*$_{\Delta SP}$, aTc-inducible expression BspC-Flag-PhoA$_{\Delta SP}$	This study
p*SP*$_{bspC}$-*phoA*$_{\Delta SP}$	Derivative of p*phoA*$_{\Delta SP}$, aTc-inducible expression SP$_{BspC}$-Flag-PhoA$_{\Delta SP}$	This study
p*ha.bspC*$_{\Delta SP}$-*phoA*$_{\Delta SP}$	Derivative of p*phoA*$_{\Delta SP}$, aTc-inducible expression BspC$_{\Delta SP}$-Flag-PhoA$_{\Delta SP}$	
p*vceA*-*phoA*$_{\Delta SP}$	Derivative of p*phoA*$_{\Delta SP}$, aTc-inducible expression VceA-Flag-PhoA$_{\Delta SP}$	This study
p*SP*$_{vceA}$-*phoA*$_{\Delta SP}$	Derivative of p*phoA*$_{\Delta SP}$, aTc-inducible expression SP$_{VceA}$-Flag-PhoA$_{\Delta SP}$	This study
p*vceA*$_{\Delta SP}$ -*phoA*$_{\Delta SP}$	Derivative of p*phoA*$_{\Delta SP}$, aTc-inducible expression VceA$_{\Delta SP}$-Flag-PhoA$_{\Delta SP}$	This study
p*bspD*-*phoA*$_{\Delta SP}$	Derivative of p*phoA*$_{\Delta SP}$, aTc-inducible expression BspD-Flag-PhoA$_{\Delta SP}$	This study
p*eipA*-*phoA*$_{\Delta SP}$	Derivative of p*phoA*$_{\Delta SP}$, aTc-inducible expression EipA-Flag-PhoA$_{\Delta SP}$	This study
p*p17*-*phoA*$_{\Delta SP}$	Derivative of p*phoA*$_{\Delta SP}$, aTc-inducible expression p17-Flag-PhoA$_{\Delta SP}$	This study
p*ttpA*-*phoA*$_{\Delta SP}$	Derivative of p*phoA*$_{\Delta SP}$, aTc-inducible expression TtpA-Flag-PhoA$_{\Delta SP}$	This study
p*hibit*	Derivative of pJS140, aTc-inducible expression Flag-HiBiT for N-terminal fusions	This study
p*bspC*-*hibit*	Derivative of p*hibit*, aTc-inducible expression BspC-Flag-HiBiT	This study
p*ha.bspC*$_{\Delta SP}$-*hibit*	Derivative of p*hibit*, aTc-inducible expression HA.BspC$_{\Delta SP}$-Flaf-HiBiT	This study
p*vceA*-*hibit*	Derivative of p*hibit*, aTc-inducible expression VceA-Flag-HiBiT	This study
p*vceA*$_{\Delta SP}$-*hibit*	Derivative of p*hibit*, aTc-inducible expression VceA$_{\Delta SP}$-Flag-HiBiT	This study
p*bspL*-*hibit*	Derivative of p*hibit*, aTc-inducible expression BspL-Flag-HiBiT	This study
p*bpe123*-*hibit*	Derivative of p*hibit*, aTc-inducible expression BPE123-Flag-HiBiT	This study
p*p17*-*hibit*	Derivative of p*hibit*, aTc-inducible expression p17-Flag-HiBiT	This study
p*eipA*-*hibit*	Derivative of p*hibit*, aTc-inducible expression EipA-Flag-HiBiT	This study

Table S3: Primers used in this study.

Primer	Sequence	Purpose
prMK197	TTGAAGCCGGCTGGCGCCAAGCTTCTCT GCAGTCCCGTCAACAAAAGCG	FW homology region 1 for Δ*virB9* from *B. abortus* 2308
prMK198	CCCGGGCGATTTGATGACCGCAAGCAGG AATCTTTTC	RV homology region 1 for Δ*virB9* from *B. abortus* 2308
prMK199	AAAAGATTCCTGCTTGCGGTCATCAAATC GCCCGG	FW homology region 2 for Δ*virB9* from *B. abortus* 2308
prMK200	AAGGCCTTGACTAGAGGGTCGACCTCGC TCGCAGAACACTTC	RV homology region 2 for Δ*virB9* from *B. abortus* 2308
prMK201	GTTGCGTGTGACCACG	Sequencing primer inside homology region 1 Δ*virB9*
prMK202	CTTGCGGATCGTCACC	Sequencing primer Δ*virB9*
prMK203	CGGTTTTGGTCGTACTACG	Sequencing primer Δ*virB9*
prMK117	GAAAAGTGAAGAGCTCATGGATTATAAAG ATCATGACGGTAGCGGTCGGACACCAGA AATGCC	FW Flag-PhoA without SP from *Escherichia coli* str. K12 substr. MG1655 for p*phoA*$_{\Delta SP}$
prMK118	CAATAACTGCCTTAAAAGCTTTTATTTCAG CCCCAGAGC	RV Flag-PhoA without SP from *Escherichia coli* str. K12 substr. MG1655 for p*phoA*$_{\Delta SP}$
prMK123	GATAGAGTTATTTTACCACTCCCTATCAG TGATAGAGAAAAGTGAAGAGCTCATGAAA CAAAGCACTATTGCAC	FW SP$_{PhoA}$ from *Escherichia coli* str. K12 substr. MG1655 for p*SP*$_{phoA}$-*phoA*$_{\Delta SP}$
prMK124	GAACAGGCATTTCTGGTGTCCGACCGCT ACCGTCATGATCTTTATAATCACCGCTAC CGGCTTTTGTCACAGGGG	RV SP$_{PhoA}$ from *Escherichia coli* str. K12 substr. MG1655 for p*SP*$_{phoA}$-*phoA*$_{\Delta SP}$
prMK151	GATAGAGTTATTTTACCACTCCCTATCAG TGATAGAGAAAAGTGAAGAGCTCATGAAA TCGACCAAGATCATACTTTC	FW *bspC* from *B. abortus* 2308 for p*bspC-phoA*$_{\Delta SP}$ and p*bspC-hibit*
prMK152	AACAGGCATTTCTGGTGTCCGACCGCTA CCGTCATGATCTTTATAATCACCGCTACC CTTGCGCACGATTTCTATG	RV *bspC* from *B. abortus* 2308 for p*bspC-phoA*$_{\Delta SP}$ and p*ha.bspC*$_{\Delta SP}$-*phoA*$_{\Delta SP}$
prMK149	GATAGAGTTATTTTACCACTCCCTATCAG TGATAGAGAAAAGTGAAGAGCTCATGAAA TCGACCAAGATCATACTTTC	FW *SP*$_{bspC}$ from *B. abortus* 2308 for p*SP*$_{bspC}$-*phoA*$_{\Delta SP}$
prMK150	AACAGGCATTTCTGGTGTCCGACCGCTA CCGTCATGATCTTTATAATCACCGCTACC GGCATCAGCTTGCGC	RV *SP*$_{bspC}$ from *B. abortus* 2308 for p*SP*$_{bspC}$-*phoA*$_{\Delta SP}$
prMK210	CACTCCCTATCAGTGATAGAGAAAAGTGA AGAGCTCATGTACCCATACGATGTTCCAG ATTACGCTGTGCCAAAGCGGACAAAG	FW *ha.bspC*$_{\Delta SP}$ from *B. abortus* 2308 for p*ha.bspC*$_{\Delta SP}$-*hibit* and p*ha.bspC*$_{\Delta SP}$-*phoA*$_{\Delta SP}$
prMK163	GATAGAGTTATTTTACCACTCCCTATCAG TGATAGAGAAAAGTGAAGAGCTCATGAAA ATCATCATCACGGCAGC	FW *vceA* from *B. abortus* 2308 for p*vceA-phoA*$_{\Delta SP}$, p*SP*$_{vceA}$-*phoA*$_{\Delta SP}$, and p*vceA-hibit*
prMK164	AACAGGCATTTCTGGTGTCCGACCGCTA CCGTCATGATCTTTATAATCACCGCTACC GTTCTTGGGCGCGTGGC	RV *vceA* from *B. abortus* 2308 for p*vceA-phoA*$_{\Delta SP}$ and p*vceA*$_{\Delta SP}$ -*phoA*$_{\Delta SP}$
prMK165	AACAGGCATTTCTGGTGTCCGACCGCTA CCGTCATGATCTTTATAATCACCGCTACC GGCCATTGCGCCCGTG	RV *SP*$_{vceA}$ from *B. abortus* 2308 for p*SP*$_{vceA}$-*phoA*$_{\Delta SP}$
prMK166	GATAGAGTTATTTTACCACTCCCTATCAG TGATAGAGAAAAGTGAAGAGCTCATGCAA AAAACCCAATGCGATGCAAAG	FW *vceA*$_{\Delta SP}$ from *B. abortus* 2308 for p*vceA*$_{\Delta SP}$-*phoA*$_{\Delta SP}$, and p*vceA*$_{\Delta SP}$-*hibit*
prMK131	GATAGAGTTATTTTACCACTCCCTATCAG TGATAGAGAAAAGTGAAGAGCTCATGATT CAATCCGCTCTCTTTTTG	FW *bspD* from *B. abortus* 2308 for p*bspD-phoA*$_{\Delta SP}$
prMK121	CTACCGTCATGATCTTTATAATCACCGCT ACCTTGCATGTCGCGGATGC	RV *bspD* from *B. abortus* 2308 for p*bspD-phoA*$_{\Delta SP}$

prMK156	GATAGAGTTATTTTACCACTCCCTATCAGTGATAGAGAAAAGTGAAGAGCTCATGAATCGATTTTTGAAGATCACTATTC	FW *bspL* from *B. abortus* 2308 for p*bspL-hibit*
prMK114	GATAGAGAAAAGTGAAGAGCTCATGAGCTTGTTGCTGGCTAAC	FW *bpe123* from *B. abortus* 2308 for p*bpe123-hibit*
prMK216	CCTTGTAATCGATGTCATGATCTTTATAATCACCGTCATGGTCTTTGTAGTCTGCCTGTCCCGCCAG	RV *bpe123* from *B. abortus* 2308 for p*bpe123-hibit*
prMK214	CACTCCCTATCAGTGATAGAGAAAAGTGAAGAGCTCATGGCCATACCATCCCTG	FW *eipA* from *B. abortus* 2308 for p*eipA-phoA*$_{\Delta SP}$, and p*eipA-hibit*
prMK278	CTACCGTCATGATCTTTATAATCACCGCTACCGAACGGGTTCCAGGTCG	RV *eipA* from *B. abortus* 2308 for p*eipA-phoA*$_{\Delta SP}$
prMK268	GATAGAGTTATTTTACCACTCCCTATCAGTGATAGAGAAAAGTGAAGAGCTCATGAACCAAAGCTGTCCG	FW *p17* from *B. abortus* 2308 for p*p17-phoA*$_{\Delta SP}$, and p*p17-hibit*
prMK276	CTACCGTCATGATCTTTATAATCACCGCTACCGACAAGCGCGGCGAT	RV *p17* from *B. abortus* 2308 for p*p17-phoA*$_{\Delta SP}$
prMK272	GATAGAGTTATTTTACCACTCCCTATCAGTGATAGAGAAAAGTGAAGAGCTCATGCGGCTAGGATCGAAAC	FW *ttpA* from *B. abortus* 2308 for p*ttpA-phoA*$_{\Delta SP}$
prMK277	CTACCGTCATGATCTTTATAATCACCGCTACCATCGGTTGTCGGCTTTTC	RV *ttpA* from *B. abortus* 2308 for p*ttpA-phoA*$_{\Delta SP}$
prMK206	GAAAAGTGAAGAGCTCATGGACTACAAAGACCATGACGGTG	FW *hibit* for p*hibit*
prMK207	CAATAACTGCCTTAAAAGCTTTTAGCTAATCTTCTTGAACAGCCG	RV *hibit* for p*hibit*
prMK208	CCTTGTAATCGATGTCATGATCTTTATAATCACCGTCATGGTCTTTGTAGTCCTTGCGCACGATTTCTATG	RV *bspC* from *B. abortus* 2308 for p*bspC-hibit* and p*ha.bspC*$_{\Delta SP}$*-hibit*
prMK252	CTTTATAATCACCGTCATGGTCTTTGTAGTCGTTCTTGGGCGCGTGGC	RV *vceA* from *B. abortus* 2308 for p*vceA-hibit* and p*vceA*$_{\Delta SP}$*-hibit*
prMK251	CTTTATAATCACCGTCATGGTCTTTGTAGTCGTTGGCCGTGCAGAAATG	RV *bspL* from *B. abortus* 2308 for p*bspL-hibit*
prMK269	CTTTATAATCACCGTCATGGTCTTTGTAGTCGACAAGCGCGGCGAT	RV *p17* from *B. abortus* 2308 for p*p17-hibit*
prMK215	CCTTGTAATCGATGTCATGATCTTTATAATCACCGTCATGGTCTTTGTAGTCGAACGGGTTCCAGGTCG	RV *eipA* from *B. abortus* 2308 for p*eipA-hibit*

Table S4: *In silico* analysis of *B. abortus* effector proteins with T4SS-dependent translocation predicted signal peptides and N-proximal transmembrane domains for a subset of putative effector proteins.

Effector	ORF[1]	Size [kDa]	SignalP6.0 [Likelihood]		Phobius		DeepTMHMM		Translocation/Secretion mechanism
			Sec/SPI (Signal Peptide)[2]	Sec/SPII (Lipoprotein Signal Peptide)[2]	Sec/SPI (Signal Peptide)[2]	N-proximal Transmembrane Domain[2,3]	N-proximal Transmembrane Domain[2,3]	Signal Peptide	
VceA	BAB1_1652	11.3	Yes	-	Yes	-	-	Yes	T4SS-dependent translocation (De Jong *et al.* 2008)
VceC	BAB1_1058	44.9	-	-	-	Yes	Yes	-	T4SS-dependent translocation (De Jong *et al.* 2008)
BPE005	BAB1_2005	16.8	-	-	-	-	-	-	T4SS-dependent translocation (Marchesini *et al.* 2011)
BPE043	BAB1_1043	166.4	-	-	-	-	-	-	T4SS-dependent translocation (Marchesini *et al.* 2011)
BPE123	BAB2_0123	16.7	-	-	Yes	-	Yes	-	T4SS-dependent translocation, periplasmic intermediate (Marchesini *et al.* 2011; Del Giudice *et al.* 2016)
BPE275	BAB1_1275	27.7	-	-	-	Yes	Yes	-	T4SS-dependent translocation (Marchesini *et al.* 2011)
SepA	BAB1_1492	21.0	-	-	-	-	-	-	T4SS-dependent translocation, periplasmic intermediate (Döhmer *et al.* 2014)
BtpA/Btp1	BAB1_0279	28.0	-	-	-	-	-	-	T4SS-dependent translocation (Salcedo *et al.* 2013)
BtpB/Btp2	BAB1_0756	31.8	-	-	-	-	-	-	T4SS-dependent translocation (Salcedo *et al.* 2013)
BspA	BAB1_0678	21.7	-	-	-	Yes	Yes	-	T4SS-dependent translocation (Myeni *et al.* 2013)
BspB	BAB1_0712	20.5	-	-	Yes	-	Yes	-	T4SS-dependent translocation (Myeni *et al.* 2013)
BspC	BAB1_0847	14.8	Yes	-	Yes	-	-	Yes	T4SS-dependent translocation (Myeni *et al.* 2013)
BspE	BAB1_1675	12.7	-	-	-	-	-	-	T4SS-dependent translocation (Myeni *et al.* 2013, 2025)
BspF	BAB1_1948	48.8	-	-	-	-	-	-	T4SS-dependent translocation (Myeni *et al.* 2013)
BspL	BAB1_1533	18.8	Yes	-	Yes	-	-	Yes	T4SS-dependent translocation (Luizet *et al.* 2021)
NyxA	BAB1_0296	14.5	-	-	-	-	-	-	T4SS-dependent translocation (Louche *et al.* 2023)
NpeA	BAB2_0195	24.4	-	Yes	Yes	-	-	Yes	T4SS-dependent translocation, periplasmic intermediate (Giménez *et al.* 2024)
RS14565	BAB2_0295	33.4	-	-	-	-	-	-	T4SS-dependent secretion, translocation into host cell (Yin *et al.* 2024)
RS15060	BAB2_1002	35.8	-	-	-	-	-	-	T4SS-dependent secretion, translocation into host cell (Yin *et al.* 2024)
RS10635	BAB2_0074	35.7	-	-	Yes	-	Yes	-	T4SS-dependent secretion, translocation into host cell (Yin *et al.* 2024)
Rhg1	BAB1_0478	33.2	-	-	-	Yes	Yes	-	T4SS-dependent translocation (Cabello *et al.* 2024)

[1] open reading frame in *B. abortus* 2308 genome
[2] Yes = Probability ≥0.9
[3] within N-proximal 50 aa

Table S5: *In silico* analysis of *B. abortus* secreted and translocated proteins with unresolved translocation or secretion mechanism predicted signal peptides and N-proximal transmembrane domains for a subset of putative effector proteins.

Effector	ORF[1]	Size [kDa]	SignalP6.0 [Likelihood]		Phobius		DeepTMHMM		Translocation/Secretion mechanism
			Sec/SPI (Signal Peptide) [2]	Sec/SPII (Lipoprotein Signal Peptide) [2]	Sec/SPI (Signal Peptide)[2]	N-proximal Transmembrane Domain[2,3]	N-proximal Transmembrane Domain[2,3]	Signal Peptide	
RicA	BAB1_1279	18.6	-	-	-	-	-	-	T4SS-dependent translocation, T4SS-independent secretion (De Barsy *et al.* 2011)
BPE159	BAB2_0159	18.5	-	-	-	Yes	Yes	-	T4SS-independent translocation (Marchesini *et al.* 2011)
BPE865/BspI	BAB1_1865	24.9	-	-	-	-	-	-	Translocated, unclear mechanism (Marchesini *et al.* 2011; Myeni *et al.* 2013)
BspD	BAB1_1611	34.9	-	-	-	Yes	Yes	-	Translocated, unclear mechanism (Myeni *et al.* 2013)
BspG	BAB1_0277	23	-	-	-	-	-	-	T4SS-independent translocation (Myeni *et al.* 2013, 2025)
BspH	BAB1_1864	53.7	-	-	-	-	-	-	Translocated, unclear mechanism (Myeni *et al.* 2013)
BspJ	BAB2_0119	19.6	-	-	-	-	-	-	Translocated, unclear mechanism (Myeni *et al.* 2013; Ma *et al.* 2020)
BspK	BAB2_0541	14.3	-	-	-	Yes	Yes	-	T4SS-independent translocation (Myeni *et al.* 2013)
NyxB	BAB1_1101	15.0	-	-	-	-	-	-	T4SS-independent secretion (Louche *et al.* 2023)
CstA	BAB2_0856	51.7	-	-	-	-	-	-	T4SS-independent secretion (De Barsy *et al.* 2012)
SodC	BAB2_0535	18.1	Yes	-	Yes	-	-	Yes	T4SS-independent secretion, periplasmic protein (Beck, Tabatabai and Mayfield 1990; Bricker *et al.* 1990; Liu *et al.* 2018)

[1] open reading frame in *B. abortus* 2308 genome
[2] Yes = Probability ≥0.9
[3] within N-proximal 50 aa

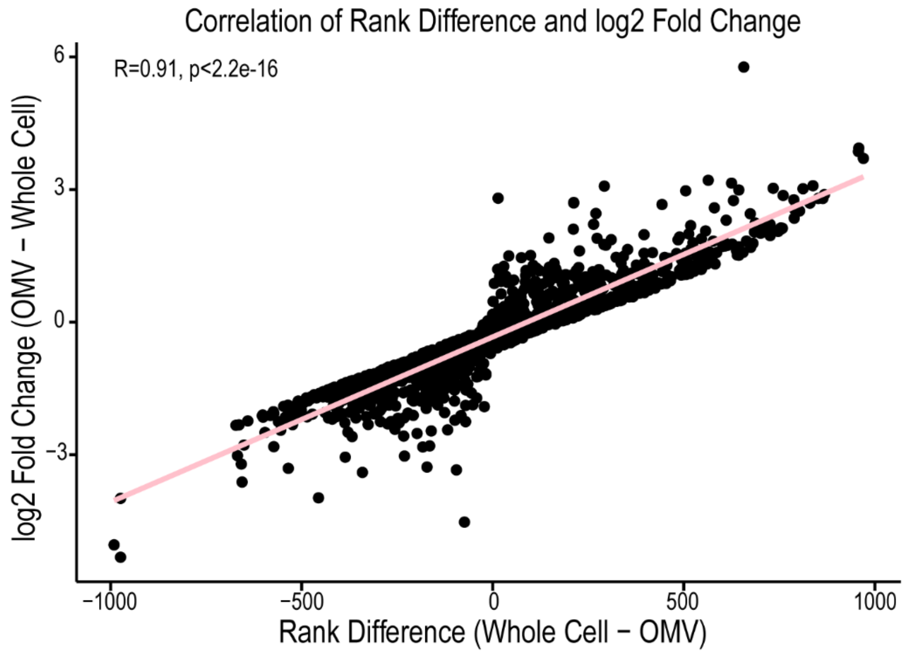

Figure S1: Correlation of rank difference between proteins quantified in whole cells and OMVs and log2 fold change of proteins quantified in OMVs vs whole cells.

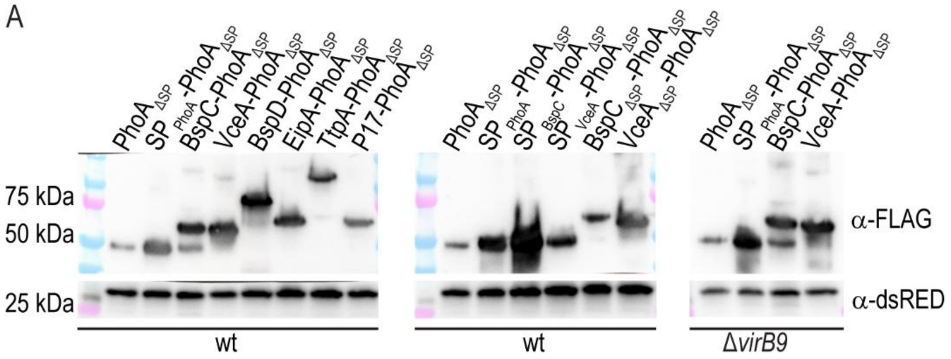

Figure S2: Western Blots to control expression of PhoA-fusion proteins. **(A)** Equal amounts of bacterial pellet were loaded onto an SDS-PAGE and visualized using Western Blotting. Shown is one representative replicate of two.

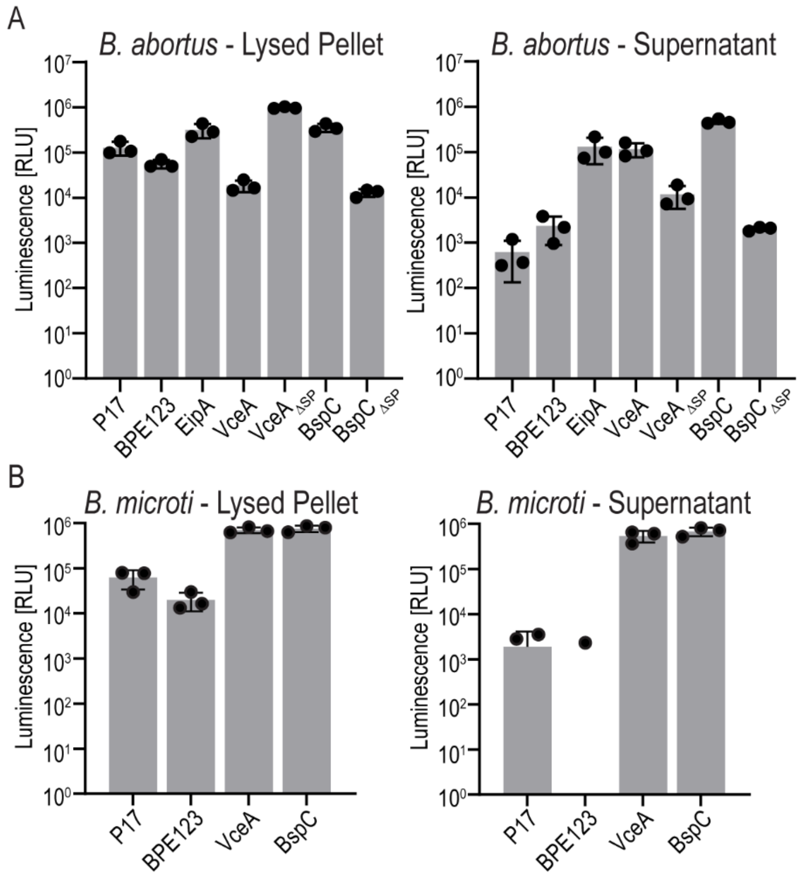

Figure S3: RAW luminescence measurements corresponding to Figure 2A and D. RLU = Relative Light Units. Log-scale. Background substracted. n=3. **(A)** Luminescence measurements from lysed *B. abortus* expressing HiBiT-fusion proteins (left) and bacteria-free supernatant (right) supplemented with LgBiT. **(B)** Luminescence measurements from lysed *B. microti* expressing HiBiT-fusion proteins (left) and bacteria-free supernatant (right) supplemented with LgBiT. In the supernatant fraction some values for P17 and BPE123 were negative after background subtraction and are therefore not blotted.

Data S1_SupplementaryData. For each identified protein, the orthogroup was generated using OrthoFinder version 2.5.5 to match *Brucella* proteins between species to compare published proteomes at the orthogroup level.

Data S2_SupplementaryData. Lists identified ions and peptides, as well as proteins from three replicates and associated search engine scores. Also listed are proteins exclusively found in the whole cell or OMV-fractions, named “WCExcl” and “OMVExcl”, respectively.

Data S3_SupplementaryData. Lists the quantified proteins from 3 replicates (“AnnotatedQuantData”) and their scores. Also listed are proteins exclusively found in the whole cell or OMV-fractions, named “WCExcl” and “OMVExcl”, respectively.

Data S4_SupplementaryData. Lists all proteins that were quantified at least once in OMVs and whole-cell samples ranked by abundance and log2 fold change. “Marker Rank Difference” lists the rank difference and log2 fold change of all marker proteins depicted in Fig. 1A and B. “T4SS Rank and FC” lists the rank difference and log2 fold change of T4SS effectors depicted in Fig. 1C.

Data S5_SupplementaryData. Shows in how many of the three replicates the listed effectors were identified and quantified.

Data S6_SupplementaryData. Lists the identified effectors and indicates their presence or absence in OMV proteomes of *B. melitensis* (Avila-Calderón *et al.* 2020), *B. abortus* (Araiza-Villanueva *et al.* 2019), and *B. suis* (Socorro Ruiz-Palma *et al.* 2021), as well as if the protein was quantified in the *B. abortus* OMVs and if it was enriched or depleted.

Supplementary References

Araiza-Villanueva M, Avila-Calderón ED, Flores-Romo L *et al.* Proteomic analysis of membrane blebs of *Brucella abortus* 2308 and RB51 and their evaluation as an acellular vaccine. *Frontiers in Microbiology* 2019;**10**.

Avila-Calderón ED, Medina-Chávez O, Flores-Romo L *et al.* Outer membrane vesicles from *Brucella melitensis* modulate immune response and induce cytoskeleton rearrangement in peripheral blood mononuclear cells. *Frontiers in Microbiology* 2020;**11**.

Beck BL, Tabatabai LB, Mayfield JE. A protein isolated from *Brucella abortus* is a Cu-Zn superoxide dismutase. *Biochemistry* 1990;**29**:372–6.

Bricker BJ, Tabatabai LB, Judge BA *et al.* Cloning, expression, and occurrence of the *Brucella* Cu-Zn superoxide dismutase. *Infect Immun* 1990;**58**:2935–9.

Cabello A-L, Wells K, Peng W *et al. Brucella*-driven host N-glycome remodeling controls infection. *Cell Host & Microbe* 2024, DOI: 10.1016/j.chom.2024.03.003.

Celli J, Salcedo SP, Gorvel JP. *Brucella* coopts the small GTPase Sar1 for intracellular replication. *Proceedings of the National Academy of Sciences* 2005;**102**:1673–8.

De Barsy M, Jamet A, Filopon D *et al.* Identification of a *Brucella* spp. secreted effector specifically interacting with human small GTPase Rab2. *Cellular Microbiology* 2011;**13**:1044–58.

De Barsy M, Mirabella A, Letesson J-J *et al.* A *Brucella abortus cstA* mutant is defective for association with endoplasmic reticulum exit sites and displays altered trafficking in HeLa cells. *Microbiology* 2012;**158**:2610–8.

De Jong MF, Sun YH, Den Hartigh AB *et al.* Identification of VceA and VceC, two members of the VjbR regulon that are translocated into macrophages by the *Brucella* type IV secretion system. *Molecular Microbiology* 2008;**70**:1378–96.

Del Giudice M, Döhmer P, Spera J *et al.* VirJ is a *Brucella* virulence factor involved in the secretion of type IV secreted substrates. *The Journal of biological chemistry* 2016;**291**:12383–93.

Döhmer PH, Valguarnera E, Czibener C *et al.* Identification of a type IV secretion substrate of *Brucella abortus* that participates in the early stages of intracellular survival. *Cellular Microbiology* 2014;**16**:396–410.

Giménez A, Del Giudice MG, López PV *et al. Brucella* NpeA is a secreted Type IV effector containing an N-WASP-binding short linear motif that promotes niche formation. Roy CR (ed.). *mBio* 2024;**15**:e00726-24.

Harms A, Segers FHID, Quebatte M *et al.* Evolutionary dynamics of pathoadaptation revealed by three independent acquisitions of the VirB/D4 Type IV Secretion System in *Bartonella*. *Genome Biology and Evolution* 2017;**9**:761–76.

Ketterer M, Chiquet P, Esposito M *et al.* The putative type 4 secretion system effector BspD is involved in maintaining envelope integrity of the pathogen *Brucella*. D'Orazio SEF (ed.). *mSphere* 2024;**9**:e00232-24.

Liu X, Zhou M, Yang Y *et al.* Overexpression of Cu-Zn SOD in *Brucella abortus* suppresses bacterial intracellular replication via down-regulation of Sar1 activity. *Oncotarget* 2018;**9**:9596–607.

Louche A, Blanco A, Lacerda TLS *et al.* Brucella effectors NyxA and NyxB target SENP3 to modulate the subcellular localisation of nucleolar proteins. *Nat Commun* 2023;**14**:102.

Luizet J-B, Raymond J, Lacerda TLS *et al.* The *Brucella* effector BspL targets the ER-associated degradation (ERAD) pathway and delays bacterial egress from infected cells. *Proc Natl Acad Sci U S A* 2021;**118**:e2105324118.

Ma Z, Li R, Hu R *et al. Brucella abortus* BspJ Is a nucleomodulin that inhibits macrophage apoptosis and promotes intracellular survival of *Brucella*. *Frontiers in Microbiology* 2020;**11**.

Marchesini MI, Herrmann CK, Salcedo SP *et al.* In search of *Brucella abortus* type IV secretion substrates: Screening and identification of four proteins translocated into host cells through VirB system. *Cellular Microbiology* 2011;**13**:1261–74.

Myeni S, Child R, Ng TW *et al. Brucella* modulates secretory trafficking via multiple type IV secretion effector proteins. *PLoS Pathogens* 2013;**9**:e1003556.

Myeni S, Child R, Ng TW *et al.* Correction: *Brucella* modulates secretory trafficking via multiple Type IV secretion effector proteins. *PLoS Pathog* 2025;**21**:e1013302.

Salcedo SP, Marchesini MI, Degos C *et al.* BtpB, a novel *Brucella* TIR-containing effector protein with immune modulatory functions. *Frontiers in Cellular and Infection Microbiology* 2013;**3**:28.

Socorro Ruiz-Palma M del, Avila-Calderón ED, Aguilera-Arreola MaG *et al.* Comparative proteomic analysis of outer membrane vesicles from *Brucella suis*, *Brucella ovis*, *Brucella canis* and *Brucella neotomae*. *Arch Microbiol* 2021;**203**:1611–26.

Yin Y, Tian M, Zhang G *et al.* Identification of Brucella RS15060 as a novel type IV secretion system effector associated with bacterial virulence. *Vet Res* 2024;**55**:168.
